# Effect of Dietary Supplemented with Mulberry Leaf Powder on Growth Performance, Serum Metabolites, Antioxidant Property and Intestinal Health of Weaned Piglets

**DOI:** 10.3390/antiox12020307

**Published:** 2023-01-28

**Authors:** Jiayu Ma, Jian Wang, Xiaoyun Jin, Sujie Liu, Shaofeng Tang, Zhenghua Zhang, Shenfei Long, Xiangshu Piao

**Affiliations:** 1 State Key Laboratory of Animal Nutrition, College of Animal Science and Technology, China Agricultural University, Beijing 100193, China; 2 MOA Key Laboratory of Animal Virology, Department of Veterinary Medicine and Center of Veterinary Medical Sciences, Zhejiang University, Hangzhou 310027, China; 3 Hubei Mulberry Biotechnology Co., Ltd., Jingzhou 434200, China

**Keywords:** mulberry leaf powder, antioxidant capacity, intestinal function, microbiota, piglets

## Abstract

**Background:** The study aimed to explore the effect of mulberry leaf powder (MP) on the performance, serum metabolites and antioxidant property, as well as intestinal health, of weaned piglets. A total of 120 healthy piglets weighing 8.43 ± 1.91 kg (Duroc × (Landrace × Yorkshire); weaned at 28 d) were chosen and classified into four treatments with three replicates of 10 piglets each based on a randomized complete block design (barrow:gilt = 1:1). The diet treatments were a corn–soybean meal basal diet added with 0% (Ctrl), 2% (MP_2), 4% (MP_4) and 6% MP (MP_6), respectively. The feeding experiment was 28 days in total. The feeding period lasted for 28 days in total. **Results:** The diet supplemented with 2% MP had no detrimental effects on the growth performance, immunity, enzyme capacity and inflammatory factors, as well as intestinal barrier function. MP_2 is capable of decreasing the levels of serum D-lactic acid and lactate dehydrogenase, enhancing the superoxide dismutase capacity in the liver and diminishing the potential pathogenic bacteria *Allisonella* in the colon. However, compared with MP_2, MP_6 had unfavorable effects on the average daily gain and average daily feed intake; the concentration of serum non-esterified fatty acids; the activities of superoxide dismutase and glutathione peroxidase and the capacity of lipase and amylase, as well as the intestinal barrier function-related mRNA expression of occludin, claudin-1 and mucin-2 in piglets. **Conclusion:** Taken together, piglets fed with 2% MP had no adverse effect and was capable of improving the serum metabolites, enhancing the antioxidant capacity (SOD) and lowering the potential pathogenic bacteria of the hindgut (*Allisonella*). However, the highest concentration of MP (6%) may cause detrimental effects for piglets, which are probably associated with the higher antinutritional factors and fiber. Therefore, the dietary supplementation of 2% MP for piglets may be advisable.

## 1. Introduction

The early weaning of piglets is a fundamental technology in the field of intensive pig production worldwide. Altogether, it has the advantages of ameliorating the reproductive performance of sows, minimizing the opportunity of disease transmission between sows and piglets, as well as increasing the production performance of piglets [1]. At the early weaning stages, the digestive and immune systems of piglets have not developed completely, combined with the impact of various stress factors such as feeding patterns and environmental changes, the gut structure and function of piglets are vulnerable to alterations, and the mortality and morbidity rates are higher, which seriously affect the economic efficiency of pig production [2,3]. Consequently, improving the growth performance and immune status of early-weaned piglets is a topical issue of current research in livestock. However, with the rapid development of the animal husbandry and the global ravages of the COVID-19 epidemic, the cooperation between enterprises has weakened, which has brought serious challenges to the distribution and supply of feed for the livestock (such as the raw feed material procurement of soybean meal, fishmeal). Hence, it is urgent to develop untraditional feed resources, which enable to effectively alleviate the shortage of conventional feed resources and contribute to lowering the production cost of feed for enterprises [4]. Recently, several researchers have confirmed that unconventional feed sources (brown rice, corn germ meal and wheat protein meal) can be considered as some alternatives to cereal concentrates in livestock feeds with no detrimental effects on performance. [5,6,7]. Furthermore, unconventional feed ingredients have a multitude of properties, the most noteworthy of which are their anti-bacterial and antioxidant properties [8].

Mulberry (*Morus alba* L.) is a fast-growing, long-lived, weed-resistant, deciduous plant with high adaptability to climate and soil, which be capable of mowing 3–4 times annually and will be available for 20–30 years with one planting. China is a large mulberry-cultivating country with plentiful mulberry resources, including 15 mulberry species and 4 varieties, and the area under mulberry cultivation in China is approximately 1.06 million hectares [9,10]. Mulberry leaves contain a large number of nutrients. Research has proven that mulberry leaves are wealthy in essential amino acids [11]. Especially, mulberry leaves have numerous bioactive substances, including flavonoids (anti-aging effects), alkaloids, mulberry furan and glycosides (anti-hypertensive), mulberry ketones (antibiotic activity), polysaccharides (antioxidant activity), morusin (anti-tumor), umbelliferone (anti-inflammatory), 1-deoxynojirimycin (DNJ, diuretic and hypoglycemic) and γ-aminobutyric acid (vascular elasticity) [12,13,14,15,16]. Further, mulberry leaves are palatable to animals and can either be directly eaten fresh or fed dried. Recently, with the Food and Agriculture Organization of the United Nations (FAO) attached higher importance to the usage of mulberry leaf resources for the expansion of animal husbandry, scholars have begun to strengthen the research concerning mulberry leaves as livestock feed [17]. China also issued the “Mulberry Leaf Powder for Feeding” (SB/T 0998-2013) in 2013, which aim to enthusiastically exploit mulberry sources, as well as extend its utilization path. Our previous findings in finishing pigs have shown that dietary supplementation with 9% mulberry leaf powder (MP) had no detrimental effects on performance, immuno-oxidant properties, carcass traits, muscle lipids and amino acids, but ameliorated the serum immunity (IgM), meat color (a * 24 h) and intestinal barrier function and increased the relative abundance of hindgut *Bifidobacteria* in finishing pigs [18]. Another study regarding intestinal digestibility in sheep revealed that the digestible energy and crude protein values of mulberry leaf were equivalent to alfalfa hay [19] and mulberry leaf is suitable as a novel feed additive to regulate the antioxidant property of laying hens [20].

Hence, the main purpose of our study is to explore the potential of MP to substitute the expensive conventional feeds (soybean-meal) and determine the appropriate concentration of MP. Meanwhile, we focused on the beneficial effect of MP on intestinal health in piglets, which might be meaningful and helpful for the future application of MP in livestock.

## 2. Materials and Methods

The study was performed at the animal base of the Ministry of Agriculture of the Feed Industry Center (Fengning, Hebei, CHINA). All animal experimental projects were accredited and authorized by the Institutional Animal Care and Use Committee of China Agricultural University (No. AW10601202–1-2, Beijing, China).

### 2.1. Mulberry Leaf Powder Product

The product mainly acquired from the leaves, buds and young branches of mulberry trees, which provided by Hubei Mulberry Biotechnology Co., Ltd. (Jinzhou, China). The approaches of harvesting were described as previously [18]. The mulberry leaves used in our experiment were collectively harvested with combine harvester at the mid-July (usually harvested 3–4 times per year). The nutrient profiles of MP were summarized in Table S1.

### 2.2. Diets and Animal Desgin

A total of 120 piglets without antibiotic injection (Duroc × [Landrace × Yorkshire]; weaned at 28 d and weighed 8.43 ± 1.91 kg) were chosen from farrowing house and classified into four treatments with three replicates of 10 piglets each based on randomized complete block design (barrow:gilt = 1:1). The diet treatments were corn–soybean meal basal diet added with 0% (Ctrl), 2% (MP_2), 4% (MP_4) and 6%MP (MP_6), respectively. The feeding period was last for 28 days in total. The diet formulation was described in Table S2, which fulfilled or exceeded the NRC [21] for piglets.

The selected piglets were positioned in 1.5 × 1.5 m^2^ pens, each pen have a slatted floor, adjustable troughs and duckbill drinkers. The nursery was thoroughly disinfected, and the troughs were cleansed before the experiment star, the piglets were administered using an all-in/all-out feeding paradigm. The air temperature (28–30 °C in the first week, then reduced by 1–2 °C per week until 24–26 °C), humidity (60–70%), CO_2_ concentration (<0.15%) and ammonia (<20 mg/m^3^) in the nursery were organized using an auto-control system. Notably, in order to alleviate the nutritional stress of piglets, the first 3 days after weaning were adjusted progressively as follows: day 1, 75% creep feed + 25% experimental feed; day 2, 50% creep feed + 50% experimental feed; day 3, 25% creep feed + 75% experimental feed. The feeding and health condition of piglets were watched and logged daily. The chosen piglets were feed and drink ad libitum during the feeding experiment. The pens were cleaned periodically, the piglets were routinely vaccinated and dewormed conforming to the protocols of the animal base. No piglet morbidity or mortality (except diarrhea) were noticed during the whole feeding experiment.

### 2.3. Performance and Diarrhea Rate

The feed intake and leftover feed of piglets were registered daily by pen for calculating the average daily feed intake (ADFI) on d 0, d 14 and d 28, and the selected piglets were individually weighted to calculate the average daily gain (ADG); then, the feed conversion ratios (FCR = ADFI/ADG) were obtained. Additionally, the chosen piglets were examined anally at 09:00 and 15:00 daily during the experiment, while the amount of piglets with diarrhea were counted, and then, the diarrhea rate was calculated using the following formula:

Diarrhea rate (%) = Number of piglets with diarrhea/(Total number of chosen piglets × Experimental days) × 100%

### 2.4. Slaughtering and Collecting

On the morning of 14d and 28d of the experiment, the bloods (approximately 10 mL from the anterior vena cava) were gathered from one piglet with an almost average body weight, respectively, stand for 3 h, centrifuged at 3000× *g* for 10 min at 4 °C, gathered the serum and storage at −20 °C for the next step.

At the end of the feeding experiment, three piglets with closely average weight were fasting for 12 h before transported to slaughter house, then slaughtered and dissected after anesthesia with sodium pentobarbital from each treatment. The anterior 1/3 segment of the duodenal, jejunal and ileal tissues (approximately 2 cm) were collected, respectively, the samples of intestine were washed off using 0.9% saline and fixed in 10% neutral formalin buffer for intestinal morphological analysis. The mucosal tissues of pancreas, jejunum and ileum were lightly scraped with a sterile scalpel and transferred into 10 mL cryotube, placed into liquid nitrogen and storage at −80 °C for next analysis of digestive enzyme activity and inflammatory factors. Meanwhile, the jejunum and ileum (approximately 2 cm) at the at the 1/3 of the posterior segment were rinsed with phosphate buffered saline, transferred into 10 mL cryotube, stored at −80 °C for mRNA expression analysis relevant to intestinal barrier function. Additionally, approximately 5 mL of ileal, cecum and colonic chyme were gathered respectively for 16 s bacterial sequencing analysis. Each sample was gathered twice (one for testing and one for backup).

### 2.5. Serological Analysis and Enzyme Activities

The serum samples were defrosted at 4 °C and mixed evenly before determination. The antioxidant indexes (superoxide dismutase, SOD; glutathione peroxidase, GSH-Px; catalase, CAT; total antioxidant capacity, T-AOC; malondialdehyde, MDA) in the serum and liver were detected by a spectrophotometry (722S, Lengguang Technology, Shanghai, China). The concentration of serum insulin-like growth factor-1 (IGF-1) were measured by radioimmunoassay by a DFM-96 radioimmunoassay counter (Zhongcheng Electromechanical Technology, Hefei, China).

The enzyme linked immunosorbent assay (ELISA) was conducted to measure the serum immunoglobulins (IgA, IgG and IgM); inflammatory factors (interleukin; tumor necrosis factor-α, TNF-α; gamma-interferon, IFN-γ); hormones (glucocorticoid, GC; adrenocorticotropic hormone, ACTH; epinephrine, EPI; epidermal growth factor, EGF) and metabolites (D-lactate, DLA; non-esterified fatty acid, NEFA), as well as the intestinal inflammatory factors and secretory immunoglobulin A (sIgA) by the Multiskan Ascent fully automated enzyme marker (Thermo, Waltham, MA, USA).

The remaining serum metabolites such as glucose (GLU), total cholesterol (TC), total triglycerides (TG), high-density lipoprotein (HDL), low-density lipoprotein (LDL), alanine aminotransferase (ALT), aspartate aminotransferase (AST), total protein (TP), albumin (ALB), globulin (GLB), alkaline phosphatase (ALP), lactate dehydrogenase (LDH), uric acid (UA) and blood urea nitrogen (BUN) were detected using a CLS880 fully automatic biochemical analyzer (Zecen Biotech, China). The activities of amylase, lipase and trypsin in the pancreas, jejunum and ileum were analyzed by immunoturbidimetry by a UV–Vis spectrophotometer (UV1100, MAPADA, Shanghai, China).

The mentioned methods involved reagents were delivered by Nanjing Jiancheng Institute of Biological Engineering (Nanjing, China), and the corresponding kits were operated strictly according to the manufacturing instructions.

### 2.6. Intestinal Morphometry

The gut samples were fixed in 4% paraformaldehyde solution for 48 h, rinsed, excised and dehydrated with ethanol for 24 h, paraffin-embedded and excised into 4 cross-sections for staining with hematoxylin–eosin. Samples were examined morphologically with an CX31 optical microscope (Olympus, Japan) in conjunction with true color image analysis software, and 4 pairs of intact, well-oriented villi and crypts were observed in each sample. The top of the villi to the junction of the villi and crypt was defined the villus height (VH), and the depth of the villi invagination reflected the crypt depth (CD). Then calculated the ratio of villus height to crypt depth (VH/CD) after the measurement.

### 2.7. Real-Time PCR

As described in the manufacturing instructions, the TRizol (Invitrogen, Waltham, MA, USA) kit was used for the extraction of total RNA from the jejunum and ileum of piglets, and the concentration of RNA was authenticated by 1% agarose gel electrophoresis (Biowest, Spain), then reverse transcribed into cDNA using a *TransScript^®^* All-in-One First-Strand cDNA Synthesis SuperMix for PCR kit (QIAGEN, Germany). Reverse transcription system: RNA, 0.5 μg; 5 × *TransScript*^®^ All-in-one SuperMix for qPCR, 5 μL; gDNA Remover, 0.5 μL; added RNase-free water until 10 μL. Reaction procedure: 42 °C, 15 min; 85 °C, 5 s added to 90 μL RNase-free water after reverse transcription. Fluorescent quantitative PCR protocols were performed by the Roche LightCycler^®^ 480II Real-Time PCR System (Roche, Switzerland; PCR efficiency: 96~102%). PCR reaction mixture system (10 μL): template (cDNA), 1 μL; 2 × *PerfectStart^TM^Green qPCR SuperMix*, 5 μL; forward primer, 0.2 μL; reverse primer, 0.2 μL; RNase-free water, 3.6 μL. Reaction system: pre-denaturation at 95 °C for 5 min; denaturation at 95 °C for 10 s, annealing at 60 °C for 30 s; elongation at 72 °C for 30 s, 40 cycles. An analysis of the melting curve was performed at the end of the PCR cycle to verify the specificity of the expected PCR product generation. The electrophoresis was carried out using 1% agarose gel at 120V for 20 min, then examined by gel imaging system (BIO-RAD, USA) after the electrophoresis. Each sample was measured thrice. The primer sequences were presented in Table S3, which was accomplished with the assistance of Beijing Tianyi Huiyuan Biotechnology (Beijing, China). Normalization of gene expression levels was conducted using housekeeping gene (β-Actin), and the relative expression was calculated on the basis of the 2^−ΔΔCt^ approach.

### 2.8. 16s rRNA Sequencing

The 16s RNA bacterial sequencing was finished in collaboration with Majorbio Bio-Pharm Co. Ltd. (Shanghai, China). In brief, the digesta of the ileum, cecum and colon were obtained from the −80 °C. The total bacterial genomic DNA was isolated from chyme samples with the E.Z.N.A.^®^ Soil DNA Kit (Omega Bio-Tek, Norcross, GA, USA) according to the instructions. Extracted DNA was detected by 1% agarose gel and a NanoDrop 2000 UV–Vis spectrophotometer (Thermo Scientific, Wilmington, NC, USA) was employed for determining DNA concentration and purity. The primers 338F (5′-ACTCCTACGGGAGGCAGCAG-3′) and 806R (5′-GGACTACHVGGGTWTCTAAT-3′) were used to amplify the hypervariable region V3-V4 of the bacterial 16S rRNA gene with ABI GeneAmp^®^ 9700 PCR thermocycler (ABI, CA, USA). The PCR reaction system (20 µL): 5 × FastPfu buffer, 4 μL; dNTPs, 2 μL; forward primer (5 μM), 0.8 μL; reverse primer (5 μM), 0.8 μL; FastPfu DNA Polymerase, 0.4 μL; bovine serum albumin (BSA), 0.2 μL; template DNA, 10 ng; finally, added RNase-free water to 20 μL. The PCR amplification procedure: pre-denaturation at 95 °C for 3 min, denaturing at 95 °C for 30 s, annealing at 55 °C for 30 s and elongation at 72 °C for 45 s, 27 cycles; stable elongation at 72 °C for 10 min and termination at 4 °C. PCR reactions were carried out in triplicate. The amplification products were extracted from 2% agarose gel, purified and recovered by the Axyprep DNA Gel Extraction kit (Axygen Biosciences, Union City, CA, USA) in accordance with the manufacturer’s instructions, then quantified by Quantus™ Fluorometer (Promega, Madison, WI, USA).

Purified amplification products were mixed equimolarly and sequenced in pairs on an Illumina MiSeq PE300 platform (Illumina, San Diego, CA, USA) following the standard protocol available at Majorbio Bio-Pharm Co. Ltd. (Shanghai, China) The source reads have been uploaded to the NCBI with accession number PRJNA890539 (Ileum), PRJNA890541 (Cecum) and PRJNA890544 (Colon).

The raw sequencing reads were demultiplexed, quality-filtered by fastp (v0.23.2, https://github.com/OpenGene/fastp, accessed on 12 October 2022) and merged by FLASH (v1.2.11, https://ccb.jhu.edu/software/FLASH/index.shtml, accessed on 16 October 2022) to obtain high-quality effective tags with reference to the tags quality control process of QIIME (V 1.9.1, http://qiime.org/install/index.html, accessed on 16 October 2022) [22]. The operational taxonomic units (OTU) were clustered by UPARSE software (v 7.1, http://drive5.com/uparse/, accessed on 21 October 2022) based on the 97% similarity cutoff, and chimeras were removed [23,24]. The taxonomy of each OTU representative sequence was examined by the RDP classifier (v 2.13, http://rdp.cme.msu.edu/, accessed on 21 October 2022) on the basis of Bayesian algorithm. Finally, the identified classifications were matched against the Silva 16S rRNA database (v138, http://www.arb-silva.de, accessed on 16 October 2022) with a 70% confidence threshold [25].

Mothur software (v1.30.2 https://mothur.org/wiki/calculators/ accessed on 22 January 2023) was employed to assess the microbial α-diversity; the principal coordinate analysis (PCoA) and similarity analysis (ANOSIM) based on the Bray–Curtis distance matrix algorithm were used for assessing bacterial β-diversity by R software; Circos were plotted by Circos mapping software (v 0.69.9, http://circos.ca/, accessed on 22 November 2022) to elucidate the relationships between the proportion of dominant species composition and group. The LEFSe analysis combined with an all-against-all multi-group comparison strategy (linear discriminant analysis score > 2.5) on the basis of the non-parametric factorization Kruskal–Wallis sum test and Wilcoxon rank sum test was applied for estimating features with significant differences in abundance and identifying taxa with significant abundances.

### 2.9. Statistical Analysis

All values were calculated on an individual basis, apart from the performance data, which were calculated with a pen basis. Source data were preliminarily processed with Excel 2016 (Microsoft, Redmond, WA, USA). The one-way ANOVA with generalized linear models and Turkey’s Kramer test were conducted using SAS 9.2 software (SAS Institute, Cary, NC, USA), in which the data of piglet diarrhea rate was checked by chi-square test. The linear and quadratic comparisons were used for establishing the dose-response effect of MP in piglets. Difference was considered as statistically significant when *p* < 0.05, and a statistically significant trend was deemed when *p* ≤ 0.10.

## 3. Results

### 3.1. Performance

As displayed in Table 1, from day 0 to day 14 of the experiment, the ADG and ADFI of piglets declined linearly (*p* < 0.05) with the increase level of MP. From day 15 to day 28 of the experiment, the ADFI and FCR of piglets reduced linearly (*p* < 0.05) with the increase level of MP. From day 0 to day 28 of the experiment, the ADG, ADFI and FCR of piglets showed a linear decrease (*p* < 0.05) with the increase level of MP. Moreover, no significant differences were noticed between the Ctrl and treatments in the diarrhea rate of piglet during the whole experiment.

### 3.2. Serum Indicators

As displayed in Table 2, neither on day 14 nor on day 28 of the experiment, significant differences were observed between the Ctrl and treatments concerning serum immunoglobulins and inflammatory factors (include proinflammatory cytokine and anti-inflammatory cytokine) of piglets.

As displayed in Table 3, on day 14 of the experiment, the level of serum DLA increased quadratically (*p* < 0.05) with the increment of MP concentration. On day 28 of the experiment, dietary supplementation of 2% MP decreased (*p* < 0.05) the level of serum LDH more than the Ctrl. Furthermore, the serum TG and NEFA enhanced linearly, and the serum BUN decreased linearly (*p* < 0.05) with the enhancement of the MP concentration.

### 3.3. Serum Hormones and Antioxidant Characteristics

**Figure 1:**
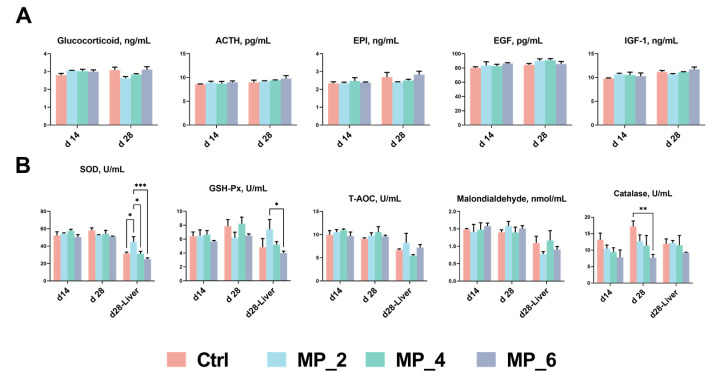
Effect of MP with various levels on serum hormone contents (**A**) and antioxidant property (**B**) in piglets. ACTH, adrenocorticotropic hormone; EPI, epinephrine; EGF, epidermal growth factor; IGF-1, insulin-like growth factor-1; SOD, superoxide dismutase; GSH-Px, glutathione peroxidase; T-AOC, total antioxidant capacity. Ctrl, MP_2, MP_4 and MP_6 basal diets containing 0%, 2%, 4% and 6% of MP, respectively. Values were presented as mean ± SEM. Bars marked with various asterisks (*) denote the grade of significant differences. *N* = 3. * *p* < 0.05; ** *p* < 0.01; *** *p* < 0.01.

As displayed in Figure 1, for serum hormones of piglets, neither on day 14 nor on day 28 of the experiment, significant differences were noted between the Ctrl and treatments on GC, ACTH, EPI, EGF and IGF-1.

For the serum antioxidant property of piglets, a decrease level (*p* < 0.05) of serum CAT was observed in MP_6 than the Ctrl. For the liver antioxidant property of piglets, the dietary supplementation of 2% MP enhanced (*p* < 0.05) the SOD activity compared with the Ctrl, MP_4 and MP_6, and MP_2 was higher (*p* < 0.05) than MP_6 in GSH-Px activity.

### 3.4. Digestive Enzyme Activity and Immunity of Intestine

**Figure 2:**
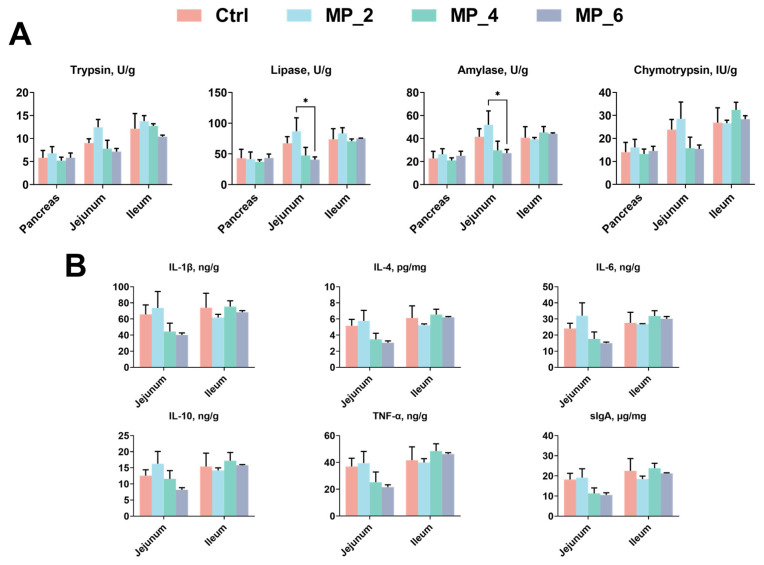
Effect of MP with various levels on digestive enzyme activity (**A**) and intestinal inflammatory factors (**B**) in piglets. IL-1β, interleukin-1β; IL-4, interleukin-4; IL-6, interleukin-6; IL-10 interleukin-10; TNF-α, tumor necrosis factor-α; sIgA, secretory immunoglobulin A. Ctrl, MP_2, MP_4 and MP_6 basal diets containing 0%, 2%, 4% and 6% of MP, respectively. Values were presented as mean ± SEM. Bars marked with various asterisks (*) denote the grade of significant differences. * *p* < 0.05. *N* = 3.

As displayed in Figure 2, for intestinal digestive enzyme activity, dietary supplementation of 2% MP ameliorated (*p* < 0.05) the lipase and amylase in jejunum of piglets than the MP_6. Nonetheless, neither pancreas nor ileum significant differences were noticed between the Ctrl and treatments on digestive enzyme activity. For intestinal inflammatory factors of jejunum and ileum in piglets, no significant differences were observed between the Ctrl and treatments on the levels of intestinal inflammatory factors and sIgA.

### 3.5. Morphology and Barrier Function of Intestine

As displayed in Table 4 and Figure S1, for intestinal morphology, no significant difference was noted concerning the villus height and crypt depth in piglets between the Ctrl and treatments.

**Figure 3:**
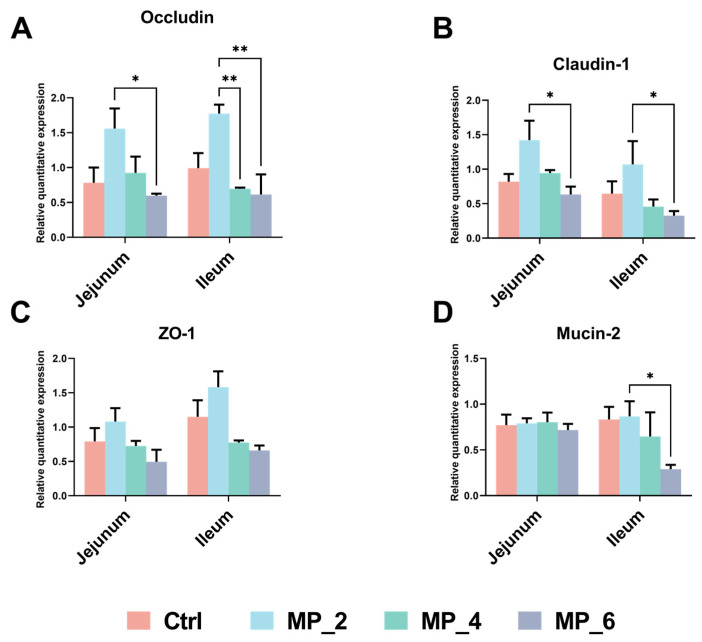
Effect of MP with various levels on mRNA expression of intestinal barrier function in piglets. (**A**) Occludin; (**B**) claudin-1; (**C**) zonula occludens-1; (**D**) mucin-2. Ctrl, MP_2, MP_4 and MP_6 basal diets containing 0%, 2%, 4% and 6% of MP, respectively. Values were prensented as mean ± SEM. Bars marked with various asterisks (*) denote the grade of significant differences. * *p* < 0.05; ** *p* < 0.01. *N* = 3.

As displayed in Figure 3, for intestinal barrier function, a dietary supplementation of 2% MP was capable of upregulating (*p* < 0.05) the mRNA expression of occludin and claudin-1 in jejunum of piglets than the MP_6 and increasing (*p* < 0.05) the mRNA expression of occludin in the ileum of piglets than the MP_4 and MP_6. Additionally, an upregulated mRNA expression (*p* < 0.05) of claudin-1 and mucin-2 were detected in the ileum of piglets supplemented with 2% MP compared with the MP_6.

### 3.6. Bacterial Sequencing and α-Diversity in Ileum, Cecum and Colon of Piglets

**Figure 4:**
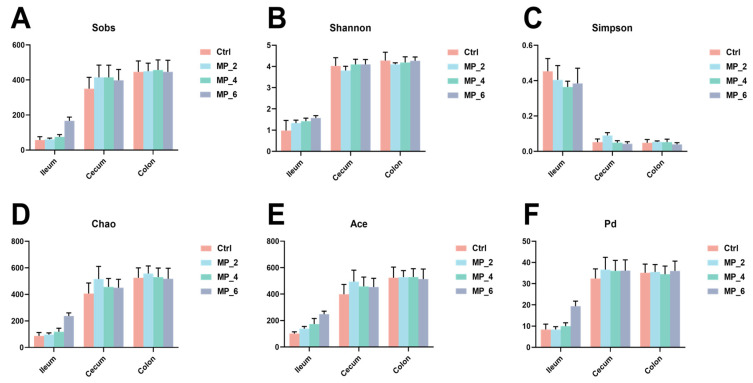
Effect of MP with various levels on bacterial α-diversity of intestine at OTU level in piglets. (**A**) Sobs index; (**B**) Shannon index; (**C**) Simpson index; (**D**) Ace index; (**E**) Chao index; (**F**) Phylogenetic diversity index. Ctrl, MP_2, MP_4 and MP_6 basal diets containing 0%, 2%, 4% and 6% of MP, respectively. Values were presented as mean ± SEM. *N* = 3.

For ileal microbiota, twelve chyme samples of piglets were sequenced and examined and a total of 757,295 optimized sequences with 420 bp average length were obtained. After a random subsample according to the smallest value of sample sequences, a total of 328 OTUs were noticed and classified into 13 phylum, 20 classes, 55 orders, 93 families, 187 genera and 273 species similarity comparison based on the Silva database. For cecal microbiota, a total of 674,635 optimized sequences with 416 bp average length were obtained, and 824 OTUs were identified and classified into 17 phylum, 29 classes, 56 orders, 87 families, 204 genera and 380 species. For colonic microbiota, a total of 861,224 optimized sequences with 417 bp average length were obtained, and 824 OTUs were identified and classified into 16 phylum, 24 classes, 46 orders, 78 families, 195 genera and 387 species, as displayed in Figure 4 and Figure S2. Nonetheless, no significant differences were noticed between the Ctrl and treatments concerning bacterial community richness (sobs, chao and ace indices) and community diversity (Shannon, Simpson and phylogenetic diversity) in the ileum, cecum and colon of piglets.

### 3.7. Bacterial Composition and β-Diversity Analysis in Ileum, Cecum and Colon of Piglets

**Figure 5:**
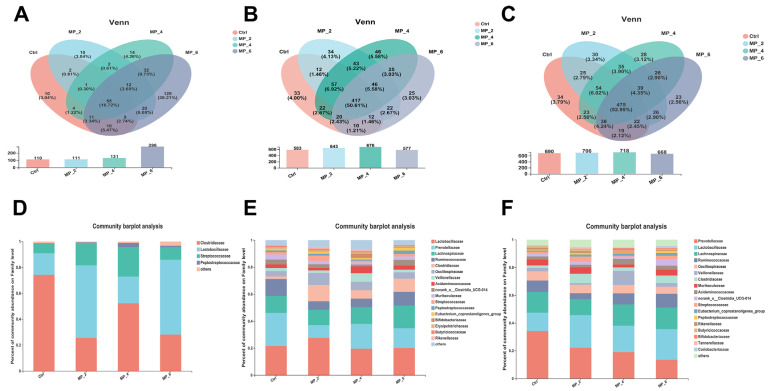
Effect of MP with various levels on gut bacterial composition in piglets. Venn analysis in ileum (**A**), cecum (**B**) and colon (**C**). Bacterial composition at family in ileum (**D**), cecum (**E**) and colon (**F**). Ctrl, MP_2, MP_4 and MP_6 basal diets containing 0%, 2%, 4% and 6% of MP, respectively. *N* = 3.

**Figure 6:**
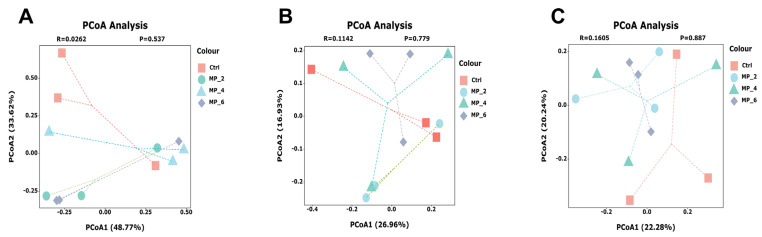
Effect of MP with various levels on bacterial β-diversity of intestine in piglets. Principal coordinate analysis (PCoA) at OTU level in the ileum (**A**), cecum (**B**) and colon (**C**). Ctrl, MP_2, MP_4 and MP_6 basal diets containing 0%, 2%, 4% and 6% of MP, respectively. *N* = 3.

**Figure 7:**
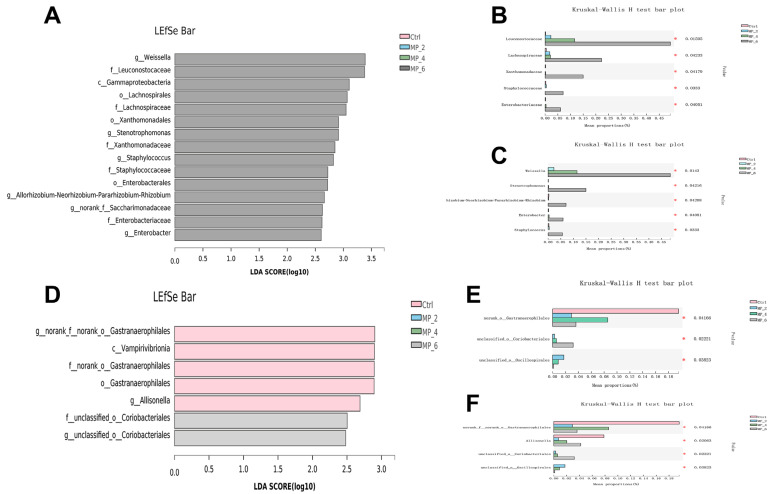
Effect of MP with various levels on the LEfSe analysis from the phylum to genus level and diagram of intestinal bacterial composition differences in piglets. (**A**) LDA in ileum; Differences in ileum microorganism at the family (**B**) and genus levels (**C**). (**D**) LDA in colon. Differences in colon microorganism at the family (**E**) and genus levels (**F**). LEfSe, linear discriminant analysis effect size; LDA, linear discriminant analysis. *p* < 0.05 and LDA score >2.5 were presented. Ctrl, MP_2, MP_4 and MP_6 basal diets containing 0%, 2%, 4% and 6% of MP, respectively. Bars marked with various asterisks (*) denote the degree of significant differences. *N* = 3. * *p* < 0.05.

For ileum of piglets, a total of 55 common OTUs, and 10 (Ctrl), 10 (MP_2), 14 (MP_4) and 129 (MP_6) unique OTUs were detected, respectively, by Venn analysis (Figure 5A). The bacterial compositions were visualized by bar plot and heatmap at the family and genus levels. The dominant microbiota at the family level (Figure 5D) in Ctrl were *Clostridiaceae* (74.40%), *Lactobacillaceae* (16.48%), *Streptococcaceae* (7.73%). The Circos plots were drawn at the family level (Figure S3) to indicate the proportion of dominant species distribution for each treatment and the proportion of each dominant species distribution for the different treatments. At the genus level (Figure S4A), the *Sarcina* (53.66%), *Clostridium_sensu_stricto_1* (20.75%), *Lactobacillus* (16.48%) and *Streptococcus* (7.72%) were dominated. For bacterial β-diversity, the result of PCoA (Figure 6A) based on the bray_curtis algorithm and ANOSIM examination indicated that there were no differences (*r* = 0.0262, *p* = 0.537) in bacterial structures among the Ctrl and treatments. Moreover, the LEfSe analysis combined with LDA examination (Figure 7A) and the results of the Kruskal–Wallis H test with Turkey’s Kramer post-hoc test (Figure 7B,C) elucidated that the ileum of piglets supplemented with 6% MP were enriched and increased (*p* < 0.05) in *Leuconostocaceae*, *Lachnospiraceae*, *Xanthomonadaceae*, *Staphylococcaceae*, *Enterobacteriaceae*, *Weissella* and *Stenotrophomonas* compared to the Ctrl, MP_2 and MP_4.

For cecum of piglets, a total of 417 common OTUs, and 33 (Ctrl), 34 (MP_2), 46 (MP_4) and 25 (MP_6) unique OTUs were detected, respectively (Figure 5B). The dominated microorganisms at the family level (Figure 5E) in Ctrl were *Prevotellaceae* (24.36%), *Lactobacillaceae* (21.69%), *Ruminococcaceae* (12.63%) and *Lachnospiraceae* (12.55%). At the genus level (Figure S4B), the *Lactobacillus* (21.69%), *Prevotella* (15.59%), *Faecalibacterium* (7.04%), *unclassified_f_Lachnospiraceae* (5.37%) and *norank_f_Muribaculaceae* were dominated. For bacterial β-diversity, the findings of PCOA (Figure 6B) showed that dietary supplemented with MP had no effect (*r* = 0.1142, *p* = 0.779) on bacterial community in cecum of piglets. The LEfse revealed that no significant difference in the relative abundance of microorganisms in the cecum of piglets between the Ctrl and treatments.

For colon of piglets, a total of 475 common OTUs, and 34 (Ctrl), 30 (MP_2), 28 (MP_4) and 23 (MP_6) unique OTUs were detected, respectively (Figure 5C). The dominant microorganisms at the family level (Figure 5F) in Ctrl were *Prevotellaceae* (33.98%), *Lactobacillaceae* (13.08%), *Lachnospiraceae* (14.90%), *Ruminococcaceae* (8.48%), *Oscillospiraceae* (6.61%) and *Muribaculaceae* (4.38%). At the genus level (Figure S4C), the *Prevotella* (24.05%), *Lactobacillus* (13.08%), *Prevotellaceae_NK3B31_group* (4.54%), *norank_f_Muribaculaceae* (4.38%) and *Agathobacter* (4.28%) were dominated. For bacterial β-diversity, the findings of PCOA (Figure 6C) presented that dietary supplemented with MP had no effect (*r* = 0.1605, *p* = 0.887) on bacterial structure in colon of piglets. Moreover, the LEfse analysis combined with LDA examination (Figure 7D) showed that the colon of piglets in Ctrl were enriched in *Vampirivibrionia*, *Gastranaerophilales* and *Allisonella* and in MP_6 was enriched in *unclassified_o_Coriobacteriales*. The results of Kruskal-Wallis H test (Figure 7E,F) elucidated that dietary supplementation of MP_2 decreased (*p* < 0.05) the abundance of *norank_o_Gastranaerophilales* at family level and *norank_f_norank_o_Gastranaerophilales* and *Allisonella* at the genus level.

## 4. Discussion

The weaning of piglets is usually correlated with the alteration of environment and diet, for example departing from the sow to commence group living and switching from breastfeeding to solid feed. The series of stresses in combination with the weaknesses of the weaned piglets such as imperfectly developed digestive and immune systems are susceptible to higher morbidity and mortality rates [26]. Additionally, the spread and worsening of the epidemic has resulted in rising prices of traditional feed ingredients, causing a lot of concern for many companies [27]. Therefore, the use of MP as an unconventional feed source to alternative the high-priced conventional feed (soybean meal, fish meal) in animal nutrition is gradually gaining attention. In the current study, dietary supplementation of MP linearly declined the ADG and ADFI from day 0 to day 14 and overall, and decreased the ADFI and FCR from day 15 to day 28. Additionally, compared with the Ctrl, MP_2 had no detrimental effects on ADG and ADFI in piglets, while MP_4 and MP_6 had greater negative effects, suggesting that diets supplemented with low level (2%) of MP might be comparable to the growth performance of the piglet in the Ctrl. A linear decrease in performance of piglets is probably attributed to the higher anti-nutritional factors such as phytic acid and tannins in MP. Phytic acid complexes with mineral components in the gastrointestinal tract could reduce the absorption and utilization of phosphorus in piglets, and is capable of binding to proteins, decreasing the functional properties and affecting the performance of piglets [28]. Tannins have the ability to react with proteins and other macromolecules for reducing nutrient utilization, which limits the application of MP in livestock. Research has revealed that excessive tannin content in plants will substantially reduce animal intake and predispose livestock to food poisoning, thus affecting the performance [29]. Furthermore, high-fiber diets are harmful for energy and nutritional absorption [30], the reduced growth performance of piglets may also be associated with high fiber content. Therefore, it is necessary to consider the reduction of fiber and antinutritional factors in MP for achieving considerable growth performance.

For further exploration of the effect of MP on piglet performance, the serums were collected on days 14 and 28 to determine the serum immunity, antioxidant properties, metabolism and hormone levels of piglets. Notably, in present study, dietary supplementation of MP had no detrimental effect on immunity of piglets, but decreased serum D-LA and LDH was observed in MP_2. D-LA is metabolized by various bacteria in the intestine and is typically used as an essential biomarker of intestinal mucosal permeability. Microbiota in the intestine proliferate when animals are in a state of hypoxia, malnutrition, stress or disease, resulting in the production of more D-LA released into the bloodstream [31]. LDH catalyzes the endoconversion of pyruvate and lactate and is responsible for the catabolism of carbohydrates, the elevated levels of LDH often reveal some disease of the liver or gallbladder [32]. Moreover, the concentrations of TC and NEFA were elevated linearly, and the concentration of BUN was declined linearly with the improvement levels of MP. Increased TC levels are a potential risk factor for the pathogenesis of coronary heart disease and some metabolic diseases [33], and NEFA levels are associated with ketone bodies, which is helpful to discern the hepatic risk [34]. The current study is similar to the results of Zhao et al. [35], who observed the higher levels of serum and liver TC, VLDL-C, and NEFA in intrauterine growth retardation (IUGR)-affected piglet. Serum BUN content is a valuable indicator of protein metabolic status and can be employed for quantifying nitrogen utilization and excretion rates [36]. The concrete mechanism underlying the reduction of serum urea nitrogen by MP is unclear, which should be further investigated.

The stimulation of piglets by environmental and feed factors after weaning induces disruption of the redox system in piglets, and the massive accumulation or lowered scavenging ability of free radicals probably leads to the detrimental effect on feed intake and growth of piglets, especially for the smaller one [37,38]. Thus, the enhanced concentration of antioxidant enzyme such as CAT, SOD and GSH-Px, as well as the lowered content of serum MDA (products of lipid peroxidation) of the piglets, are helpful to alleviate the weaning stress [19]. In present study, the diet supplemented with 2% MP exhibited a superior SOD and GSH-Px activity in the liver, whereas CAT activity was dramatically decreased in the serum of piglets fed with 6% MP, suggesting that 2% MP could modify the antioxidant function of piglets and the higher concentrations of MP might be adversely affect piglets. The antioxidant effect of MP is correlated with the active elements. Studies have proved that the polysaccharides, polyphenols and flavonoids contained in mulberry leaves not only have powerful antioxidant properties, and but also can directly scavenge superoxide ion radicals and lipid peroxides [39,40], especially the hydroxyl radicals that cannot be scavenged by enzymes, which are the primary components of mulberry leaves to exert antioxidant activity [41,42]. Overall, MP_2 showed a favorable effect on piglet serum metabolism and antioxidant capacity, while high concentrations of MP had a detrimental effect.

The integrity of the gut morphological structure is fundamental to the maintenance of normal gut function. The atrophy of the intestinal villi or increased depth of the crypt reflects a weakened capacity of the gut to absorb the nutrients [43]. Research has revealed that weaning stress damages the intestinal mucosa and supplies a substrate for pathogenic bacteria to colonize, enhancing the possibilities of adhesion and invasion. The toxins and metabolites yielded by the pathogenic bacteria are capable of disrupting the intestinal barrier (characterized by elevated gut permeability), lowering the activity of intestinal digestive enzymes, and limiting the digestion and absorption of nutrients, causing to diminished growth performance of piglets [44]. Therefore, an intact gut mucosal barrier is paramount to ensure the provision of competent nutrients to the organism [45]. Mechanical barriers, such as claudin-1, occludin, and *ZO-1*, are considerate as primary components of tight junctions and crucial regulators of paracellular permeability, as well as mucin-1, and mucin-2 secreted by goblet cells, have an essential function in regulating intestinal inflammation, which are commonly assessed for the integrity of the intestinal barrier [46]. Motivated by this, we explored further the effects of MP on intestinal morphology, digestive enzyme activity, as well as intestinal barrier function, of piglets. In present study, compared with the MP_6, dietary supplemented with 2% MP ameliorated the lipase and amylase in jejunum of piglets and upregulated the mRNA expression of occludin, claudin and mucin-2. Additionally, dietary supplemented with MP neither harmful for intestinal morphology, nor affects for the intestinal inflammatory factors, indicating that MP does not work through the immune pathway, but rather by augmenting intestinal barrier function in piglets, which is in line with our previous study in finishing pigs [18]. Additionally, the reduced digestive enzyme activity in piglets fed with 6% MP responded to the results of downregulation of intestinal barrier function. However, Horng et al. [47] has shown that polyphenols in mulberry leaves not only modulate the inflammatory response by downregulating the production of inflammatory cytokines and pro-inflammatory mediators, but also enhance the immune function by inhibiting the inflammatory response involved in the activation of macrophages mediated by the nuclear transcription factor-κB (NF-κB) signaling pathway. Therefore, a consideration is necessary to further optimize and extract the active substances (polyphenols, flavonoids) from mulberry leaves to ameliorate piglet health, even if the production cost increases.

Undeniably, the microorganisms have a determining role in the physiological and health conditions of the body. Researchers have demonstrated that the higher diversity and richness of the gut microbes, the stronger its ability to resist invasion by foreign pathogens [48,49]. In present study, dietary supplementation of MP has not affected either bacterial α-diversity or β-diversity in ileum, cecum and colon of piglets, which is in line with our previous study concerning finishing pigs, suggesting that dietary supplementation with mulberry leaf powder does not change the bacterial structure and abundance. However, dietary supplemented with 6% MP could increase the relative abundance of *Staphylococcaceae*, *Enterobacteriaceae* and *Stenotrophomonas*, these microorganisms correlate with the severe liver disease and the progression of endotoxemia [50,51]. The results were response to the findings of elevated TC and NEFA levels in serum of piglets. Meanwhile, dietary supplemented with MP_2 decreased the relative abundance of *Allisonella,* who is greater correlated with higher inflammation scores [52] and usually existence in patients with nonalcoholic steatohepatitis [53]. In brief, supplementation of MP in the diet has no detrimental effect on the intestinal microorganisms of piglets, while a high concentration of MP probably contributes to the proliferation of hazardous bacteria, thus causing inflammatory diseases.

## 5. Conclusions

In summary, dietary supplemented with 2% MP had no detrimental effects on performance, serum immunity and hormone, digestive enzyme activity and intestinal morphology and barrier function. Moreover, MP_2 is capable of improving the serum metabolites (decreased D-LA and LDH), increasing the antioxidant capacity (SOD) and lowering the potential pathogenic bacteria of hindgut (*Allisonella*). The higher concentration of MP (6%) showed a negative effect for piglets, which probably associated with the higher antinutritional factors and fiber.

The present experiment highlights the effect of MP in weaned piglets, but cannot effectively assure its positive effect during the lactation and growth periods. Moreover, the composition of MP needs to be further optimized and purified, especially the content of antinutritional factors. Hence, next we will consider optimizing the composition of MP, to reduce the negative effects and performing about 6 months feeding experiment of pigs (from birth to slaughter, approximately 1.0 kg to 100 kg) to comprehensively evaluate the effects of MP and the feasibility of replacing conventional feed in pigs. At least certainly in present study, dietary supplemented with 2% MP in piglets might be feasible.

## Figures and Tables

**Table 1:** Effect of MP with various levels on the performance and diarrhea rate in piglets.

Item	Mulberry Leaf Powder				SEM	*p*-Value		
	0	2%	4%	6%		ANOVA	Linear	Quadratic
**D 0 to 14**								
ADG, kg/d	394 ^a^	389 ^a^	358 ^ab^	328 ^b^	13.00	0.04	0.01	0.37
ADFI, kg/d	558 ^a^	553 ^a^	495 ^ab^	451 ^b^	17.24	0.01	0.01	0.30
FCR	1.41	1.42	1.38	1.38	0.02	0.17	0.06	0.59
Diarrhea rate, %	3.27	2.68	1.19	5.06	1.56	0.44	0.60	0.20
**D 15 to 28**								
ADG, kg/d	585	590	555	533	19.48	0.23	0.07	0.51
ADFI, kg/d	928	912	858	813	32.89	0.15	0.03	0.68
FCR	1.59	1.54	1.55	1.51	0.02	0.10	0.03	0.72
Diarrhea rate, %	1.79	0.59	1.57	1.79	0.47	0.31	0.65	0.18
**D 0 to 28**								
ADG, kg/d	490 ^a^	490 ^a^	456 ^ab^	430 ^b^	8.44	0.01	0.01	0.17
ADFI, kg/d	743 ^a^	732 ^a^	677 ^b^	632 ^b^	13.15	0.01	0.01	0.25
FCR	1.51	1.50	1.48	1.46	0.01	0.14	0.03	0.91
Diarrhea rate, %	2.53	1.64	1.40	3.42	0.69	0.25	0.46	0.08

^a,b^ Values with various superscripts in a row were significant differences (*p* < 0.05).

**Table 2:** Effect of MP with various levels on serum immunity in piglets.

Item	Mulberry Leaf Powder				SEM	*p*-Value		
	0	2%	4%	6%		ANOVA	Linear	Quadratic
**D 14**								
IgA, μg/mL	12.74	13.55	14.79	13.85	0.65	0.27	0.17	0.23
IgG, mg/mL	6.76	6.33	6.79	6.97	0.25	0.39	0.36	0.27
IgM, μg/mL	5.84	6.00	5.85	5.95	0.27	0.96	0.86	0.91
IL-1β, ng/L	103	104	104	103	3.45	0.99	0.93	0.85
IL-6, ng/L	33.62	31.54	32.49	33.46	1.27	0.65	0.93	0.28
IL-8, ng/L	64.32	68.66	64.46	65.95	2.28	0.54	0.95	0.55
IL-10, ng/L	18.86	19.27	19.37	18.93	0.90	0.97	0.94	0.65
TNF-α, ng/L	49.97	51.52	52.13	52.04	1.49	0.73	0.34	0.60
IFN-γ, pg/mL	143	145	149	149	4.22	0.67	0.29	0.78
**D 28**								
IgA, μg/mL	14.02	14.93	15.80	14.71	0.58	0.28	0.30	0.13
IgG, mg/mL	6.93	7.27	7.42	8.12	0.29	0.12	0.06	0.55
IgM, μg/mL	6.38	5.79	5.48	6.20	0.24	0.14	0.47	0.04
IL-1β, ng/L	102	109	105	109	4.83	0.67	0.46	0.66
IL-6, ng/L	32.05	35.88	36.35	35.24	0.93	0.06	0.05	0.04
IL-8, ng/L	69.70	69.10	65.25	75.23	3.49	0.34	0.45	0.18
IL-10, ng/L	19.69	20.37	20.25	20.13	0.62	0.87	0.68	0.54
TNF-α, ng/L	54.93	54.31	55.11	55.05	2.63	1.00	0.92	0.92
IFN-γ, pg/mL	165	156	153	170	5.57	0.22	0.65	0.06

**Table 3:** Effect of MP with various levels on serum metabolite in piglets.

Item	Mulberry Leaf Powder				SEM	*p*-Value		
	0	2%	4%	6%		ANOVA	Linear	Quadratic
**D 14**								
GLU, mmol/L	5.95	5.47	5.87	5.11	0.47	0.59	0.35	0.78
TC, mmol/L	1.69	1.61	2.25	1.99	0.32	0.53	0.33	0.79
TG, mmol/L	0.50	0.48	0.77	0.50	0.11	0.29	0.59	0.29
HDL, mmol/L	0.45	0.55	0.68	0.64	0.07	0.22	0.07	0.36
LDL, mmol/L	1.40	1.26	1.69	1.56	0.17	0.40	0.29	0.98
ALT, U/L	65.86	48.29	43.05	62.09	11.37	0.49	0.76	0.16
AST, U/L	53.10	54.45	51.55	45.90	6.07	0.77	0.40	0.58
TP, g/L	47.09	48.05	50.52	49.88	2.45	0.74	0.36	0.75
ALB, g/L	20.68	23.01	23.78	22.65	1.88	0.70	0.46	0.39
GLB, g/L	28.26	24.80	27.11	27.39	2.36	0.76	0.98	0.46
ALP, U/L	238	242	218	217	45.85	0.97	0.69	0.97
LDH, U/L	586	602	496	488	38.33	0.17	0.06	0.76
UA, μmol/L	35.39	32.67	34.11	35.04	1.34	0.52	0.95	0.22
BUN, mmol/L	1.93	1.48	2.45	1.51	0.57	0.61	0.91	0.68
DLA, μmol/L	6.68 ^a^	6.11 ^b^	6.54 ^a^	6.64 ^a^	0.11	0.04	0.58	0.02
NEFA, μmol/L	157	152	154	154	6.80	0.97	0.88	0.70
**D 28**								
GLU, mmol/L	6.40	6.17	5.67	6.44	0.45	0.62	0.86	0.31
TC, mmol/L	1.54	1.71	2.32	2.04	0.27	0.28	0.13	0.44
TG, mmol/L	0.38	0.39	0.62	0.58	0.06	0.08	0.03	0.71
HDL, mmol/L	0.49	0.61	0.76	0.60	0.10	0.38	0.33	0.21
LDL, mmol/L	1.25	1.38	1.69	1.52	0.19	0.45	0.23	0.46
ALT, U/L	56.05	48.12	48.85	45.51	5.37	0.58	0.25	0.68
AST, U/L	75.47	48.98	69.09	42.29	9.10	0.12	0.10	0.99
TP, g/L	51.28	48.32	52.59	52.12	2.02	0.49	0.48	0.56
ALB, g/L	23.98	21.70	23.73	23.37	1.80	0.81	0.98	0.61
GLB, g/L	26.34	26.16	27.18	26.58	1.48	0.96	0.80	0.89
ALP, U/L	316	285	286	226	30.93	0.31	0.10	0.66
LDH, U/L	636 ^a^	390 ^b^	547 ^ab^	429 ^ab^	50.04	0.04	0.08	0.25
UA, μmol/L	26.79	35.19	28.84	27.44	3.65	0.42	0.80	0.23
BUN, mmol/L	2.84	1.69	1.61	1.51	0.31	0.07	0.03	0.15
DLA, μmol/L	6.59	7.12	6.88	7.00	0.18	0.28	0.26	0.29
NEFA, μmol/L	161 ^b^	165 ^ab^	170 ^ab^	186 ^a^	4.83	0.04	0.02	0.27

^a,b^ Values with various superscripts in a row were significant differences (*p* < 0.05). *N* = 3.

**Table 4:** Effect of MP with various levels on the morphological analysis of intestines in piglets.

Item	Mulberry Leaf Powder				SEM	*p*-Value		
	0	2%	4%	6%		ANOVA	Linear	Quadratic
**Duodenum**								
Villus height	370	363	305	308	44.60	0.64	0.89	0.23
Crypt depth	312	339	420	369	47.23	0.47	0.28	0.44
VH/CD	1.22	1.07	0.77	0.84	0.18	0.43	0.48	0.16
**Jejunum**								
Villus height	376	460	420	543	62.61	0.36	0.15	0.76
Crypt depth	324	253	305	282	24.11	0.28	0.52	0.35
VH/CD	1.19	1.85	1.38	2.03	0.26	0.18	0.13	0.98
**Ileum**								
Villus height	213	372	293	313	33.13	0.07	0.06	0.76
Crypt depth	191	213	267	221	23.67	0.25	0.23	0.20
VH/CD	1.12	1.75	1.12	1.42	0.29	0.40	0.29	0.70

## Data Availability

The original manuscript of this study is included in the article and further information is available upon reasonable request to the corresponding author.

## Supplementary Materials

The following supporting information can be downloaded at: https://www.mdpi.com/article/10.3390/antiox12020307/s1, Table S1: Nutrient composition of MP (%, as-fed basis); Table S2: Composition and nutrient levels of basal diet (as-fed basis, %); Table S3: Primer sequences of housekeeping and target genes concerned with intestinal barrier function; Figure S1: The photomicrograph of intestinal morphology in piglets dietary supplementation of MP; Figure S2: The rarefaction curves of ileum, cecum and colon at OTU level in piglets dietary supplementation of MP; Figure S3: The Circos diagram at family level in ileum, cecum and colon of piglets; Figure S4: The heatmap analysis at genus level in ileum, cecum and colon of piglets.

## Abbreviations

MP, mulberry leaf powder; SEM, standard error of the mean; ADG, average daily gain; ADFI, average daily feed intake; FCR, feed conversion ratios; IgA, G, M, immunoglobulin A, G, M; IL-1β, 6, 8, 10, interleukin 1β, 6, 8, 10; TNF-α, tumor necrosis factor-α, IFN-γ, interferon-γ; GLU, glucose; TC, total cholesterol; TG, total triglycerides; HDL, high density lipoprotein; LDL, low density lipoprotein; ALT, alanine aminotransferase; AST, aspartate aminotransferase; TP, total protein; ALB, albumin; GLB, Globulin; ALP, alkaline phosphatase; LDH, lactate dehydrogenase; UA, uric acid; BUN, blood urea nitrogen; DLA, D-lactate; NEFA, non-esterified fatty acids; VH, villus height; CD, crypt depth; VH/CD, the ratio of villus height to crypt depth; ACTH, adrenocorticotropic hormone; EPI, epinephrine; EGF, epidermal growth factor; IGF-1, insulin-like growth factor-1; SOD, superoxide dismutase; GSH-Px, glutathione peroxidase; T-AOC, total antioxidant capacity; sIgA, secretory immunoglobulin A; OTU, Operational taxonomic units; PCoA, principal coordinate analysis; LEfSe, linear discriminant analysis effect size; LDA, linear discriminant analysis.
