# Supplementary material for: Effect of Dietary Supplemented with Mulberry Leaf Powder on Growth Performance, Serum Metabolites, Antioxidant Property and Intestinal Health of Weaned Piglets
Source: Antioxidants (Basel). 2023 Jan 28;12(2):307. doi: 10.3390/antiox12020307 (PMC9952558; doi:10.3390/antiox12020307)
Supplement: Supplementary file 1 [file antioxidants-12-00307-s001.zip › antioxidants-2129844-Supplementary material.pdf]

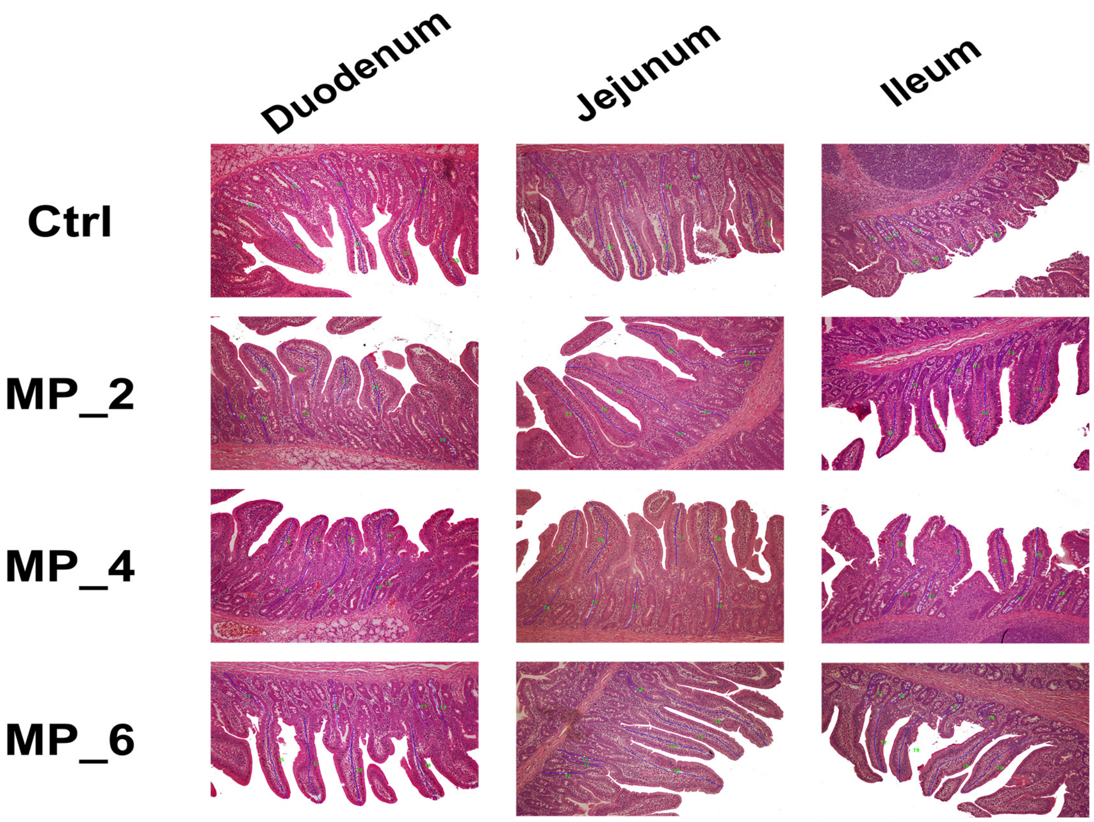

Figure S1. The photomicrograph of intestinal morphology in piglets dietary supplementation of mulberry leaf powder with varying levels. Ctrl, basal diet with 0% MP; MP_2, MP_4, MP_6, the basal diet supplemented with 2%, 4% and 6% MP, respectively. The images were visualized at 100x magnification and four pairs of villi and crypt were highlighted with blue color. *N* = 3.

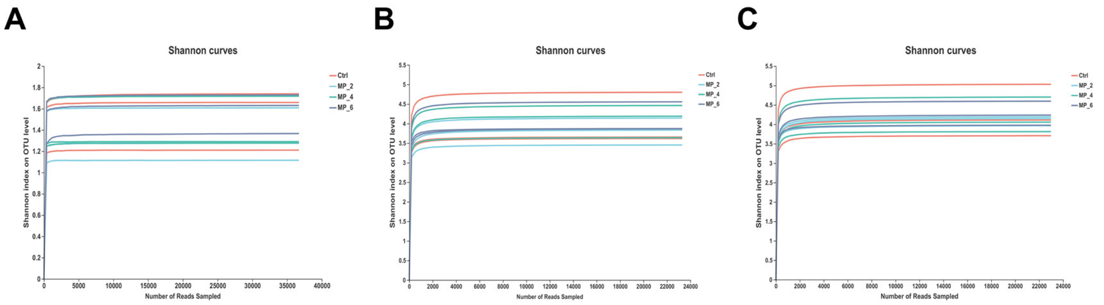

Figure S2. The rarefaction curves of ileum (A), cecum (B) and colon (C) at OTU level in piglets dietary supplementation of mulberry leaf powder with varying levels. All samples of ileum, cecum and colon indicated that the diversity indices increased sharply and eventually flatten, suggesting that the sequencing data were substantial enough for reflecting the majority of microbial diversity information in the samples.

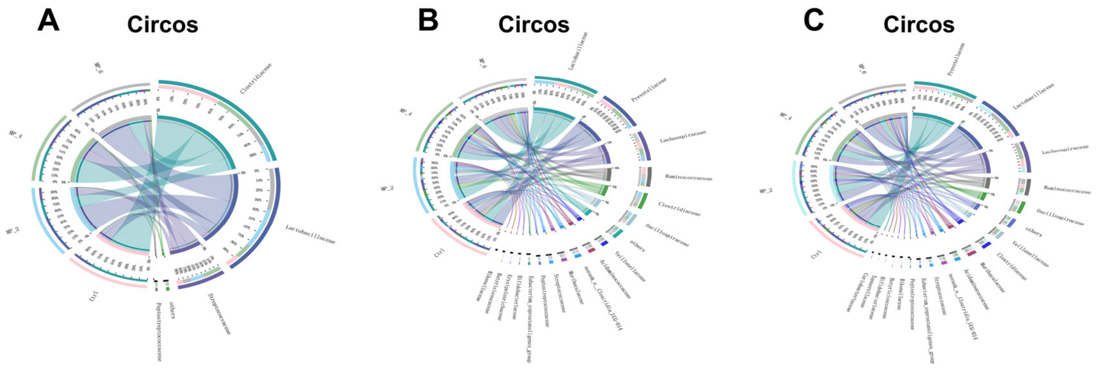

Figure S3: The Circos diagram at family level in ileum(A), cecum (B) and colon (C) of piglets. Ctrl, MP_2, MP_4 and MP_6, basal diets containing 0%, 2%, 4% and 6% of MP, respectively. N=3.

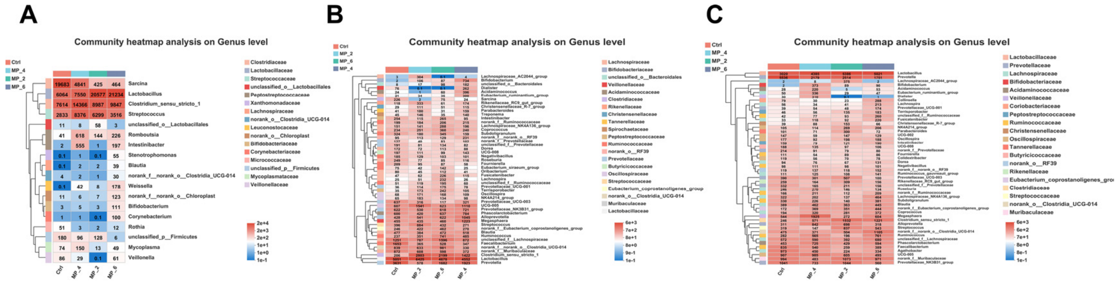

Figure S4: The heatmap analysis at genus level in ileum (A), cecum (B) and colon (C) of piglets. Ctrl, MP_2, MP_4 and MP_6, basal diets containing 0%, 2%, 4% and 6% of MP, respectively. N=3.

Table S1. Nutrient composition of mulberry leaf powder (%, as-fed basis)

Item	Mulberry powder
Dry matter	94.23
Crude protein	19.64
Ether extract	5.20
Crude fiber	34.44
Neutral detergent fiber	34.90
Acid detergent fiber	21.60
Organic matter	89.97
Gross energy	17.39
Calcium	1.64
Phosphorus	0.26
Aspartic acid	1.12
Glutamic acid	1.35
Threonine	0.51
Serine	0.46
Proline	0.49

Glycine	0.60
Alanine	0.66
Cysteine	0.07
Valine	0.64
Methionine	0.03
Isoleucine	0.51
Leucine	0.95
Tyrosine	0.32
Phenylalanine	0.58
Histidine	0.54
Lysine	0.80
Arginine	0.55
Tryptophan	0.10

Table S2. Composition and nutrient levels of basal diet (as-fed basis, %)

Item	Ctrl	2% MP	4% MP	6% MP
Corn, 8.2% CP	64.11	62.43	60.72	59.04
Soybean meal, 46% CP	9.80	9.50	9.20	8.90
Fish meal, 64.7% CP	2.50	2.50	2.50	2.50
Mulberry leaf powder	0.00	2.00	4.00	6.00
Peanut meal, 50.5% CP	3.00	3.00	3.00	3.00
Corn gluten meal, 55.6% CP	5.00	5.00	5.00	5.00
Whey powder, 3.8% CP	10.00	10.00	10.00	10.00
Soybean oil	1.00	1.00	1.00	1.00
Limestone	0.80	0.80	0.80	0.80
Dicalcium phosphate	0.85	0.85	0.87	0.87
Salt	0.33	0.33	0.33	0.33
L-lysine HCl, 78%	0.72	0.70	0.69	0.67
L-methionine, 98%	0.31	0.32	0.32	0.33
Threonine, 98%	0.33	0.32	0.32	0.31
Tryptophan, 98%	0.12	0.12	0.12	0.12
Valine	0.18	0.18	0.18	0.18
Zinc oxide	0.20	0.20	0.20	0.20
Chromic oxide	0.25	0.25	0.25	0.25
Non-antibiotic premix[1]	0.50	0.50	0.50	0.50
Total	100.00	100.00	100.00	100.00
**Nutritional levels (calculated)**				
Digestive energy, MJ/kg	14.85	14.86	14.87	14.88
Crude protein	17.78	17.71	17.64	17.56
Digestible lysine	1.31	1.31	1.31	1.31
Digestible methionine	0.63	0.63	0.63	0.63
Digestible threonine	0.94	0.94	0.94	0.94

Digestible tryptophan	0.27	0.27	0.27	0.27
Calcium	0.76	0.76	0.76	0.76
Total phosphorus	0.57	0.57	0.57	0.57
**Nutritional levels (analyzed)**				
Gross energy, MJ/kg	16.26	16.17	16.25	16.20
Crude protein	18.08	17.81	17.75	17.73
Ether extract	3.10	3.08	3.15	3.39
Dry matter	88.66	88.36	88.67	88.88
Neutral detergent fiber	8.92	9.76	12.51	12.65
Acid detergent fiber	2.57	3.75	3.82	5.22
Organic matter	94.90	94.80	94.80	94.30

CP, curde protein

[1] Non-antibiotic premix for per kilogram diet included: vitamin A, 12,000 IU; vitamin D_3, 2000 IU; vitamin E, 24 IU; vitamin K_3, 2.0 mg; vitamin B_1, 2.0 mg; riboflavin, 6.0 mg; vitamin B_6, 3 mg; vitamin B_{12}, 24 μg; nicotinic acid, 30 mg; pantothenic acid, 20 mg; folic acid, 3.6 mg; biotin, 0.1 mg; choline chloride, 0.4 mg; iron (from $FeSO_4·7H_2O$), 96 mg; copper (from $CuSO_4·5H_2O$), 8.0 mg; zinc ($ZnSO_4·H_2O$), 120 mg; manganese ($MnSO_4·H_2O$), 40 mg; iodine (from KI), 0.56 mg; selenium (from Na_2SeO_3), 0.4 mg.

Table S3. Primer sequences of housekeeping and target genes concerned with intestinal barrier function.

Item	Sequences (5' to 3')	Base	Length (bp)	T_m (℃)
occludin	F: ATGCTTTCTCAGCCAGCGTA	20	176	60
	R: AAGGTTCCATAGCCTCGGTC	20		
claudin-1	F: CAAAACCTTCGCCTTCCAG	19	293	60
	R: TCCCCACATTCGAGATGATTAC	22		
ZO-1	F: GAGGATGGTCACACCGTGGT	20	169	60
	R: GGAGGATGCTGTTGTCTCGG	20		
mucin-2	F: CGGCTCTCCAGTCTACTCGTCTAA	24	204	60
	R: TGGTTGTCGGGCAAGTTGATGA	22		
β-actin	F: TACGCCAACACGGTGCTGTC	20	207	60
	R: GTACTCCTGCTTGCTGATCCACAT	25		

F, forward primer; R, Reverse primer

ZO-1 = zonula occludens-1
